# Okara-enriched fermented sorghum instant porridge: effects on mineral bioaccessibility and structural properties

**DOI:** 10.3389/fnut.2025.1690627

**Published:** 2025-10-23

**Authors:** Adeyemi Ayotunde Adeyanju, Oluwafemi Ayodeji Adebo

**Affiliations:** ^1^Centre for Innovative Food Research (CIFR), Department of Biotechnology and Food Technology, Faculty of Science, University of Johannesburg, Johannesburg, Gauteng, South Africa; ^2^Department of Food Science and Nutrition, College of Pure and Applied Sciences, Landmark University, Omu-Aran, Kwara State, Nigeria

**Keywords:** sorghum, okara, fermentation, instant porridges, mineral bioaccessibility

## Abstract

This study investigated the impact of okara inclusion and fermentation on bioaccessible minerals and structural properties of sorghum-based instant porridges. Fermented and unfermented porridges were formulated using sorghum, okara, and their blends (70:30 and 50:50). Mineral composition and bioaccessibility, as well as structural characterization, including scanning electron microscopy (SEM), X-ray diffraction (XRD), and Fourier transform infrared spectroscopy (FTIR), were analyzed. Okara inclusion elevated Fe and Zn contents, while fermentation improved their bioaccessibility. Blends with okara were richer in iron than their 100% sorghum counterparts by approximately 6–9% and 15–47% for fermented and unfermented porridge samples, respectively. Bioaccessible Fe increased by up to 194%, and Zn reached 32% compared to sorghum-only porridges. SEM revealed gelatinization and enzymatic breakdown, while XRD and FTIR confirmed altered starch crystallinity and molecular interactions. Specifically the FTIR spectra showed that the amide I and amide II bands, approximately 1,642 cm^−1^ and 1,513 cm^−1^, associated with C=O stretching and N–H bending of proteins, respectively, were stronger in porridge samples containing okara, which aligns with its higher protein content compared to sorghum These results highlight the potential of okara-fortified fermented sorghum porridge to deliver improved nutrition and foster circular food systems by transforming an agro-industrial by-product into a valuable ingredient.

## 1 Introduction

Micronutrient deficiencies, often called “hidden hunger,” continue to be a major global concern, especially in low- and middle-income countries where diets are anchored on cereal staples (1). Essential minerals, including iron, zinc, calcium, and magnesium, are vital for growth, immune health, and metabolism. However, their absorption from plant-based sources is often limited due to antinutritional substances like phytates, tannins, and polyphenols (2, 3). Consequently, dietary and processing approaches that increase the bioaccessibility of these nutrients from conventional staples are essential for both reducing the micronutrient gap and achieving more sustainable food systems.

Sorghum (*Sorghum bicolor* L. Moench) is a drought-adapted cereal consumed across sub-Saharan Africa and Asia, providing a substantial source of dietary energy. Its ability to flourish in low-rainfall, poor-soil environments, coupled with contributions of carbohydrates, dietary fiber, and bioactive phytochemicals, makes it a staple of nutritional and agricultural importance (4–6). Nonetheless, like other cereals, it typically has low to moderate mineral levels, with absorption impeded by intrinsic antinutritional compounds (7). Furthermore, the protein quality of sorghum-based foods is generally poor, primarily due to their low digestibility, particularly after wet cooking, and the inherently low lysine content, which is approximately 2 g per 100 g of protein (8). Practices rooted in local tradition, such as fermentation, can reduce phytate concentrations, increase nutrient bioavailability, and enhance the functional qualities of cereal products. Among these, fermented sorghum porridge stands out as both a culturally valued staple and a promising platform for micronutrient fortification.

Okara, the fibrous residue left after soybean processing, has drawn fresh interest as a nutritious raw material for modern food innovation. Rather than heading straight for landfills, this ingredient brims with high-quality plant protein, soluble and insoluble fiber, other important nutrients such as lipids, minerals, and vitamins, as well as a collection of bioactive compounds that may contribute to improved nutritional outcomes (8, 9). Its incorporation into cereal-based products aligns with global sustainability efforts by valorizing agro-industrial by-products while simultaneously enhancing food quality. Previous studies have demonstrated the feasibility of using okara to fortify baked goods (10, 11), beverages (12, 13), and extruded snacks (14, 15). However, limited information exists on the role of okara in improving mineral bioaccessibility and modifying the structural characteristics of fermented cereal-based foods such as instant porridge. This study seeks to fill this gap by examining the effects of okara enrichment and fermentation on the mineral bioaccessibility and structural properties of sorghum-based instant porridge. Understanding how okara enrichment influences mineral content, bioaccessibility, and the structural properties of sorghum-based porridges is essential for developing functional, sustainable, and culturally acceptable foods.

Consequently, the present work explores how incorporating okara influences the mineral profile, mineral bioaccessibility, and microstructure of fermented sorghum instant porridge, employing scanning electron microscopy (SEM), X-ray diffraction (XRD), and Fourier-transform infrared spectroscopy (FTIR) to analyze the samples. Outcomes of the investigation should clarify the nutritional and functional advantages of okara-fortified sorghum porridge, thereby guiding the formulation of sustainable, micronutrient-rich food matrices tailored to groups prone to mineral deficiencies. In many at-risk communities, substantial quantities of okara are produced through small-scale soymilk enterprises, yet this nutrient-dense by-product is frequently discarded or diverted to low-value uses such as animal feed. Harnessing okara as a fortificant for sorghum porridges thus presents a dual opportunity: enhancing the nutritional profile of a staple food consumed by vulnerable populations while simultaneously advancing circular bioeconomy practices through the valorization of agro-industrial waste.

## 2 Materials and methods

The soybean and red non-tannin sorghum used in this study were purchased at a local market in Bodija, Ibadan, Oyo State, Nigeria, and kept dry and cool at −10 °C in an airtight container until needed. Every reagent that was employed in the studies was of analytical grade.

### 2.1 Preparation of sorghum flour

The red, non-tannin sorghum grains were extensively cleansed to eliminate any undesirable substances before being milled into flour. The process of milling sorghum into whole-grain sorghum flour was done with a hammer mill (Christy Turner Ltd., Suffolk, United Kingdom) with a 500-μm sieve. To maintain the quality of the flour until it was used again, it was promptly sealed in an airtight plastic container and kept at −10 °C.

### 2.2 Preparation of okara flour

The process described by Adeyanju et al. (8) was followed in the production of okaara flour. Fresh okara was dried at 40 °C in a hot air oven (DHG 9053, Genlab Instrument Co., Cheshire, United Kingdom) for 48 h. A laboratory hammer mill (Christy Turner Ltd., Suffolk, United Kingdom) was equipped with a 500-μm screen to finely grind the dry material. The resultant flour was kept at −10 °C in an airtight plastic container.

### 2.3 Preparation of composite flours

Composite flours were formulated by blending sorghum and okara flours in two different weight ratios: 70:30 and 50:50, respectively. These specific proportions were chosen based on favourable outcomes from preliminary evaluations. Blends containing less than 30% okara showed minimal improvement in nutritional value, while those with more than 50% were less preferred due to the pronounced beany flavour of okara (8). The flour mixtures were thoroughly homogenized using a Kenwood mixer (KMM710, Havant, United Kingdom) set at speed 5 for 3 min.

### 2.4 Instant porridge preparation

Fermented and unfermented instant porridges were prepared by mixing the flour samples with deionized water, following the procedure outlined by Adeyanju et al. (8). The resulting porridges were freeze-dried and then ground using a blender (Silver Crest Health Food Machine, Daventry, United Kingdom) to achieve a uniform particle size that passed through a 500-μm sieve.

### 2.5 Minerals analysis

The wholegrain flour samples were digested using a nitric-perchloric acid mixture following the procedure described by Zasoski and Burau (16). The resulting digests were analyzed for iron, zinc, calcium, magnesium, phosphorus, and copper using an inductively coupled plasma-optical emission spectrometer (ICP-OES) (Spectro Analytical Instruments, Kleve, Germany). Elemental determinations were performed at emission wavelengths of 259.941 nm (Fe), 213.856 nm (Zn), 396.847 nm (Ca), 279.553 nm (Mg), 766.491 nm (K), 177.495 nm (P), and 324.754 nm (Cu).

### 2.6 *In vitro* mineral bioaccessibility

The porridge formulations were subjected to *in vitro* digestion to simulate human gastric and intestinal processes. The *in vitro* dualizability method described by Miller et al. (17) and Adeyanju et al. (7) was employed. Pepsin (P-7000), pancreatin (P-1750), and bile extract (B-8631) (Sigma-Aldrich, Johannesburg, South Africa) were used as the digestive enzymes and bile salts. Dialysis was performed using Spectra/Por 7 dialysis tubing (Ø = 20.4 mm) with a molecular weight cut-off of 10 kDa (G.I.C. Scientific, Johannesburg, South Africa). The mineral content of the dialysates was quantified by ICP-OES as previously described, omitting the digestion step. Mineral bioaccessibility (%) was calculated as the proportion of the mineral concentration in the dialysate relative to the total mineral content in the digest.

### 2.7 Scanning electron microscopy

Scanning electron micrographs of the porridge samples were acquired following the methodology outlined by Mudau and Adebo (18). The samples were mounted onto aluminum stubs and coated with a thin carbon film to enhance conductivity. Subsequently, the prepared samples were transferred to the scanning electron microscopy (SEM) chamber and analyzed using a TESCAN VEGA 3 XMU scanning electron microscope (Brno, Czech Republic). Micrographs were captured at a magnification of 5.00 kx.

### 2.8 X-ray diffraction analysis

X-ray diffraction (XRD) analysis was performed using a Bruker D8 Advance X-ray diffractometer (Bruker, Karlsruhe, Germany) and monochromatic Cu-Kα1 radiation (*λ* = 1.54 Å) (19). Scans were conducted over a range of 10° to 90°. The resulting diffractograms were analyzed using Origin software (OriginLab Corporation, Northampton, MA, United States).

### 2.9 Fourier transmission infrared analysis

The functional groups present in the porridge samples were characterized using Fourier Transform Infrared (FTIR) spectroscopy (19). The analysis was performed using a Perkin.

Elmer Spectrum 2 (PerkinElmer SA (Pty) Ltd., Midrand, South Africa), operating in the wavenumber range of 4,000 to 400 cm^−1^. Flour samples were prepared and placed on the sample holder of the instrument. Spectra were acquired and processed to identify characteristic absorption peaks corresponding to the molecular functional groups.

### 2.10 Statistical analysis

The data were expressed as mean ± standard deviation of replicate measurements. Statistical significance was assessed using one-way analysis of variance (ANOVA), and mean differences were determined using Fisher’s least significant difference (LSD) test. All statistical analyses were performed using IBM SPSS Statistics software (version 22; IBM Corp., Armonk, NY, United States). In addition, principal component analysis (PCA) was performed using SIMCA multivariate data analysis software (version 18, Umetrics AB, Umeå, Sweden) to explore patterns and visualize clustering within the dataset.

## 3 Results and discussion

### 3.1 Mineral contents

Table 1 shows the concentration of different minerals (in mg/100 g dry weight basis) in the unfermented and fermented porridge samples made from sorghum and okara, either alone or blended (70:30 and 50:50, respectively). The minerals assessed, including iron (Fe), zinc (Zn), calcium (Ca), magnesium (Mg), phosphorus (P), and copper (Cu), play important roles in the body and are crucial for different biochemical functions.

**Table 1:** Mineral contents (mg/100 g dry basis) of porridge samples from sorghum, okara, and their composites.

Minerals	USP (100%)	UOP (100%)	SOUP (70:30)	SOUP (50:50)	FSP (100%)	FOP (100%)	SOFP (70:30)	SOFP (50:50)
Fe	8.44^a^ ± 0.08	10.29^ab^ ± 0.11	8.92^a^ ± 0.45	9.18^ab^ ± 0.02	9.60^ab^ ± 0.03	19.85^d^ ± 0.20	11.03^b^ ± 0.23	14.09^c^ ± 1.27
Zn	3.77^b^ ± 0.08	3.31^a^ ± 0.06	3.51^ab^ ± 0.11	3.40^ab^ ± 0.01	4.70^c^ ± 0.08	5.23^d^ ± 0.03	4.89^cd^ ± 0.18	5.04^cd^ ± 0.16
Ca	48.57^a^ ± 1.13	167.74^e^ ± 1.95	92.85^b^ ± 4.24	110.16^c^ ± 2.94	58.34^a^ ± 2.32	278.24^f^ ± 4.81	115.66^c^ ± 1.98	154.88^d^ ± 4.24
Mg	155.16^c^ ± 4.24	89.09^a^ ± 2.26	112.55^b^ ± 3.96	102.37^ab^ ± 2.26	97.33^ab^ ± 3.39	177.67^d^ ± 7.35	110.59^b^ ± 3.08	139.43^c^ ± 5.94
P	38.12^a^ ± 2.60	46.65^a^ ± 1.98	70.17^bc^ ± 1.98	63.12^b^ ± 0.85	44.21^a^ ± 4.53	114.30^d^ ± 5.37	67.50^b^ ± 1.13	80.08^c^ ± 0.85
Cu	0.62^a^ ± 0.03	0.80^c^ ± 0.00	0.74^bc^ ± 0.02	0.73^bc^ ± 0.03	0.59^a^ ± 0.01	1.27^d^ ± 0.01	0.66^ab^ ± 0.01	0.74^bc^ ± 0.03

Values are presented as means ± standard deviation from replicate determinations (*n* = 3). Mean values within the same row sharing the same superscript letter are not significantly different (*p* > 0.05). USP, unfermented sorghum porridge; UOP, unfermented okara porridge; SOUP, sorghum okra unfermented porridge; FSP, fermented sorghum porridge; FOP, fermented okara porridge; SOFP, sorghum okara fermented porridge.

The iron content differed significantly (*p* < 0.05) among the samples. Fermented okara porridge exhibited the highest iron concentration (19.85 ± 0.20 mg/100 g), more than twice that of fermented sorghum porridge (9.60 ± 0.03 mg/100 g). The lowest iron level was observed in unfermented sorghum porridge (8.44 ± 0.08 mg/100 g). Blends with okara were always richer in iron than their 100% sorghum counterparts (by approximately 6–9% and 15–47% for fermented and unfermented porridge samples, respectively). Fermentation, in particular, was found to positively impact iron content for all the formulations, as exemplified in the sorghum:okara (50:50) blend, which had an iron content of 9.18 ± 0.02 mg/100 g, which was elevated to 14.09 ± 1.27 mg/100 g after fermentation. The increase in iron content observed following fermentation can be attributed to microbial metabolism during fermentation, which can contribute directly to the mineral profile, as certain fermenting microorganisms possess the capacity to synthesize or concentrate trace elements, including iron, within the food matrix. Another possible explanation relates to the leaching of minerals from the fermentation vessels or equipment into the fermenting substrate, a phenomenon that has been reported particularly when traditional containers made of iron or other mineral-rich materials are used (20, 21). Furthermore, the reduction in dry matter due to microbial metabolism can lead to a relative concentration effect, resulting in higher apparent mineral levels per unit weight of the fermented product. Collectively, these processes may account for the higher levels of iron detected in the fermented porridges compared with their unfermented counterparts (7, 20).

The observed zinc levels of the samples ranged between 3.31 and 5.23 mg/100 g, with fermented okara porridge having the highest concentration and unfermented okara porridge having the lowest. Zinc content increased during fermentation for all the porridge samples, with 100% okara porridge showing the greatest increase (from 3.21 ± 0.08 to 5.23 ± 0.03 mg/100 g). This finding is consistent with earlier suggestions that the increase in mineral content after fermentation might be linked to the potential synthesis of minerals by microorganisms during the process (22). There was considerable variation in the calcium content of the samples, with fermented okara porridge having the maximum calcium concentration of 278.24 ± 4.81 mg/100 g, while unfermented sorghum porridge contained the lowest amount of calcium at 48.57 ± 1.13 mg/100 g. The addition of okara was observed to increase calcium in both the unfermented and fermented samples markedly. For example, the sorghum:okara (50:50) unfermented porridge and sorghum:okara (50:50) fermented porridge had calcium contents of 110.16 ± 2.94 and 154.88 ± 4.24 mg/100 g, respectively, which were much higher than values attributed to porridge samples from sorghum only in both cases (48.57 ± 1.13 and 58.34 ± 2.32 mg/100 g for unfermented and fermented porridges, respectively). The observed magnesium levels ranged between 89.09 to 177.67 mg/100 g. Unlike calcium, unfermented sorghum porridge had higher magnesium than unfermented okara porridge, with values of 155.16 ± 4.24 mg/100 g and 89.09 ± 2.26 mg/100 g, respectively, which indicates that sorghum may be considered a better source of magnesium compared with okara. Nevertheless, the fermentation process of okara significantly elevated its magnesium content (fermented okara porridge: 177.67 ± 7.35 mg/100 g), even outperforming the unfermented sorghum sample. A parallel increase was noted in the composite fermented blends, possibly resulting from either microbial activity leading to mineral synthesis or from mineral leaching from the fermentation vessel during fermentation (20). These combined mechanisms underscores the complex interplay of environmental factors and microbial activity in enhancing nutritional profile during fermentation. Also, a reduction in dry matter due to microbial metabolism can lead to a relative concentration effect, resulting in higher apparent mineral levels per unit weight of the fermented product. Surprisingly, there was a significant reduction in the magnesium content of fermented sorghum-only porridge (97.33 ± 3.39 mg/100 g) compared with its unfermented counterparts (155.16 ± 4.24 mg/100 g). This may be attributed to the incomplete digestion of the sample during the acid digestion process, possibly resulting in the precipitation of the mineral as an insoluble salt.

Phosphorus’s content was measured between 38.12 and 114.30 mg/100 g. Fermented okara porridge was the highest, while unfermented sorghum porridge was the lowest. Blends fell between these two extremes, and fermentation was noted to enhance the concentration of phosphorus. This could be attributed to either microbial activity leading to mineral synthesis or mineral leaching from the fermentation vessel during fermentation. In comparison with the other minerals, copper levels were rather low, between 0.59 and 1.27 mg/100 g. Consistent with the other minerals, fermented okara porridge had the highest Cu level, with fermented sorghum porridge having the lowest. In general, both the inclusion of okara and fermentation significantly altered the mineral composition of the porridge samples.

### 3.2 Mineral bioaccessibility

The porridge samples showed variation in mineral bioaccessibility based on the type of raw material used, whether sorghum, okara, or a blend, as well as the processing method (fermented and unfermented). To gain insight into the influence of matrix composition and fermentation, both the absolute amount (mg/100 g) and the percentage (%) of bioaccessible minerals were measured, as shown in Table 2.

**Table 2:** Total (mg/100 g) and percentage (%) mineral bioaccessibilities of porridge samples (dry basis).

Minerals	USP (100%)	UOP (100%)	SOUP (70:30)	SOUP (50:50)	FSP (100%)	FOP (100%)	SOFP (70:30)	SOFP (50:50)
Fe	0.22^a^ ± 0.01 (2.59^a^ ± 0.04)	0.45^d^ ± 0.00 (4.40^b^ ± 0.08)	0.28^b^ ± 0.01 (3.10^a^ ± 0.22)	0.32^c^ ± 0.00 (3.43^ab^ ± 0.03)	0.35^c^ ± 0.01 (3.67^ab^ ± 0.06)	1.49^g^ ± 0.01 (7.51^c^ ± 0.13)	0.77^e^ ± 0.01 (6.95^c^ ± 0.09)	1.03^f^ ± 0.02 (7.36^c^ ± 0.82)
Zn	0.46^a^ ± 0.01 (12.14^a^ ± 0.09)	0.44^a^ ± 0.01 (13.25^a^ ± 0.07)	0.44^a^ ± 0.00 (12.39^a^ ± 0.30)	0.44^a^ ± 0.01 (12.90^a^ ± 0.34)	0.72^b^ ± 0.00 (15.32^b^ ± 0.20)	0.98^d^ ± 0.01 (18.65^c^ ± 0.10)	0.80^c^ ± 0.02 (16.36^b^ ± 0.09)	0.95^d^ ± 0.01 (18.86^c^ ± 0.73)
Ca	4.53^a^ ± 0.35 (9.33^a^ ± 0.51)	27.86^e^ ± 0.88 (16.61^d^ ± 0.33)	11.99^bc^ ± 0.71 (12.91^c^ ± 0.17)	11.06^b^ ± 1.06 (10.05^ab^ ± 1.23)	7.12^a^ ± 0.18 (12.21^bc^ ± 0.79)	26.22^e^ ± 1.41 (9.42^a^ ± 0.35)	14.49^cd^ ± 0.71 (12.52^c^ ± 0.40)	16.45^d^ ± 0.35 (10.63^abc^ ± 0.52)
Mg	32.85^a^ ± 1.89 (21.16^a^ ± 0.64)	37.57^a^ ± 1.86 (42.15^d^ ± 1.01)	33.88^a^ ± 1.61 (30.09^b^ ± 0.37)	36.89^a^ ± 0.71 (36.04^c^ ± 0.11)	45.45^b^ ± 2.30 (46.68^e^ ± 0.73)	48.14^b^ ± 1.24 (27.13^b^ ± 1.82)	55.87^c^ ± 1.93 (50.52^f^ ± 0.33)	58.05^c^ ± 1.24 (41.65^d^ ± 0.89)
P	2.96^a^ ± 0.05 (7.78^a^ ± 0.39)	3.40^a^ ± 0.12 (7.28^a^ ± 0.04)	7.13^c^ ± 0.16 (10.17^b^ ± 0.06)	5.21^b^ ± 0.02 (8.26^a^ ± 0.08)	5.58^b^ ± 0.18 (12.66^c^ ± 0.90)	17.33^f^ ± 0.21 (15.17^d^ ± 0.53)	8.83^d^ ± 0.18 (13.08^c^ ± 0.48)	11.29^e^ ± 0.53 (14.09^cd^ ± 0.51)
Cu	0.11^b^ ± 0.01 (18.47^b^ ± 0.01)	0.28^f^ ± 0.00 (35.00^d^ ± 0.44)	0.18^cd^ ± 0.01 (23.98^c^ ± 0.22)	0.19^d^ ± 0.01 (23.62^c^ ± 0.31)	0.09^a^ ± 0.01 (14.71^a^ ± 1.30)	0.25^e^ ± 0.00 (19.46^b^ ± 0.09)	0.17^c^ ± 0.00 (25.44^c^ ± 0.67)	0.19^d^ ± 0.01 (26.22^c^ ± 1.72)

Values are presented as means ± standard deviation from replicate determinations (*n* = 3). Values without the parentheses are the amount of bioaccessible minerals in mg/100 g, while the values within the parentheses are the percentage bioaccessible minerals. Mean values within the same row sharing the same superscript letter are not significantly different (*p* > 0.05). USP, unfermented sorghum porridge; UOP, unfermented okara porridge; SOUP, sorghum okra unfermented porridge; FSP, fermented sorghum porridge; FOP, fermented okara porridge; SOFP, sorghum okara fermented porridge.

All the samples showed increased bioaccessibility of Fe due to fermentation, and fermented okara porridge (100%) showed the highest Fe content of 1.49 mg/100 g, followed by sorghum:okara (50:50) fermented porridge at 1.03 mg/100 g, both showing significant improvements over their unfermented equivalents. Sorghum:okara (50:50) fermented porridge and fermented okara porridge (100%) also displayed the highest Fe bioaccessibility percentages at 7.36 and 7.51%, respectively, as compared to only 2.59% in unfermented sorghum porridge (100%). This is likely due to the fermentation process breaking down antinutritional factors like phytates that bind to Fe and lower its solubility, thus increasing its bioaccessibility (7, 20). The results further revealed that the incorporation of okara into sorghum-based composite porridges led to a marked improvement in bioaccessible iron in both unfermented and fermented samples. Compared to the sorghum-only porridges, the addition of okara resulted in a 27–45% increase in bioaccessible iron in unfermented samples and a 120–194% increase in fermented samples, highlighting the nutritional quality of okara. The enhanced bioaccessible iron observed in sorghum-okara instant porridge may be attributed to the protein- and peptide-rich composition of okara, which can function as natural chelating agents, thereby improving the solubility of iron. In particular, protein-mineral interactions involving amino acids such as histidine, cysteine, and lysine are known to stabilize iron in soluble complexes, facilitating its absorption (23, 24).

Likewise, the fermentation process notably enhanced Zn bioaccessibility. Fermented okara porridge (100%) and sorghum:okara (50:50) fermented porridge had the highest Zn levels (0.98 and 0.95 mg/100 g, respectively) and bioaccessibility percentages (18.65 and 18.86%, respectively). Fermented porridges significantly surpassed the Zn bioaccessibility levels of unfermented porridges, as shown in Table 2. The increase of Zn solubility with fermentation is likely due to the enzymatic breakdown of the food matrix and the cleavage of zinc-binding ligands, which enhances the release of zinc during digestion (20, 25). The okara-based formulations had much higher calcium levels compared to the sorghum-only samples. Unfermented okara porridge (100%) and fermented okara porridge (100%) had 27.86 and 26.22 mg/100 g of bioaccessible calcium, respectively, underlining the abundance of this mineral in okara. However, the fermented okara porridge value of 9.42% for Ca bioaccessibility is lower than the 16.61% of unfermented okara porridge, suggesting fermentation in this case reduced the percentage calcium bioavailability, plausibly due to competition with other cations like magnesium, or reprecipitation of calcium as insoluble salts. Sorghum:okara (70:30) and sorghum:okara (50:50) blended fermented porridges had moderate bioaccessible calcium values of 14.49 and 16.45 mg/100 g, with bioaccessibility percentages of 12.52 and 10.63%.

Across all samples, Mg had the highest bioaccessibility. Sorghum:okara (50:50) fermented porridge exhibited the highest amount of bioaccessible Mg (58.05 mg/100 g), while the highest percentage of bioaccessible Mg was exhibited by sorghum:okara (70:30) fermented porridge (50.52%). This suggests greater Mg bioaccessibility during microbial metabolism and enzymatic activity. These results demonstrate the importance of fermentation in improving Mg solubility, likely due to the fermentation process breaking down cell walls and phytic acid (26). Fermented samples also had higher phosphorus (P) content and bioaccessibility than unfermented samples. Fermented okara porridge (100%) had the highest P content of 17.33 mg/100 g, while sorghum:okara (50:50) followed with 11.29 mg/100 g. Fermented sorghum porridge (100%) also performed better with 12.66% and sorghum:okara (50:50) fermented porridge with 14.09% phosphorus bioaccessibility. Unfermented okara porridge maintained the highest Cu bioaccessibility at 35.00% and gradually decreased in the fermented blends, sorghum:okara 70:30 (25.44%) and 50:50, 26.22%. Fermentation of 100% sorghum porridge reduced the percentage Cu bioaccessibility to 14.71%, which was lower than that of unfermented sorghum porridge (18.47%), suggesting microbial fermentation possibly caused the formation of insoluble Cu complexes that lower bioaccessibility (27, 28). Nonetheless, the combination of sorghum with okara seemed to enhance both the quantity and percentage bioavailability of copper (Cu).

### 3.3 Principal component analysis of mineral composition and bioaccessibility

**Figure 1:**
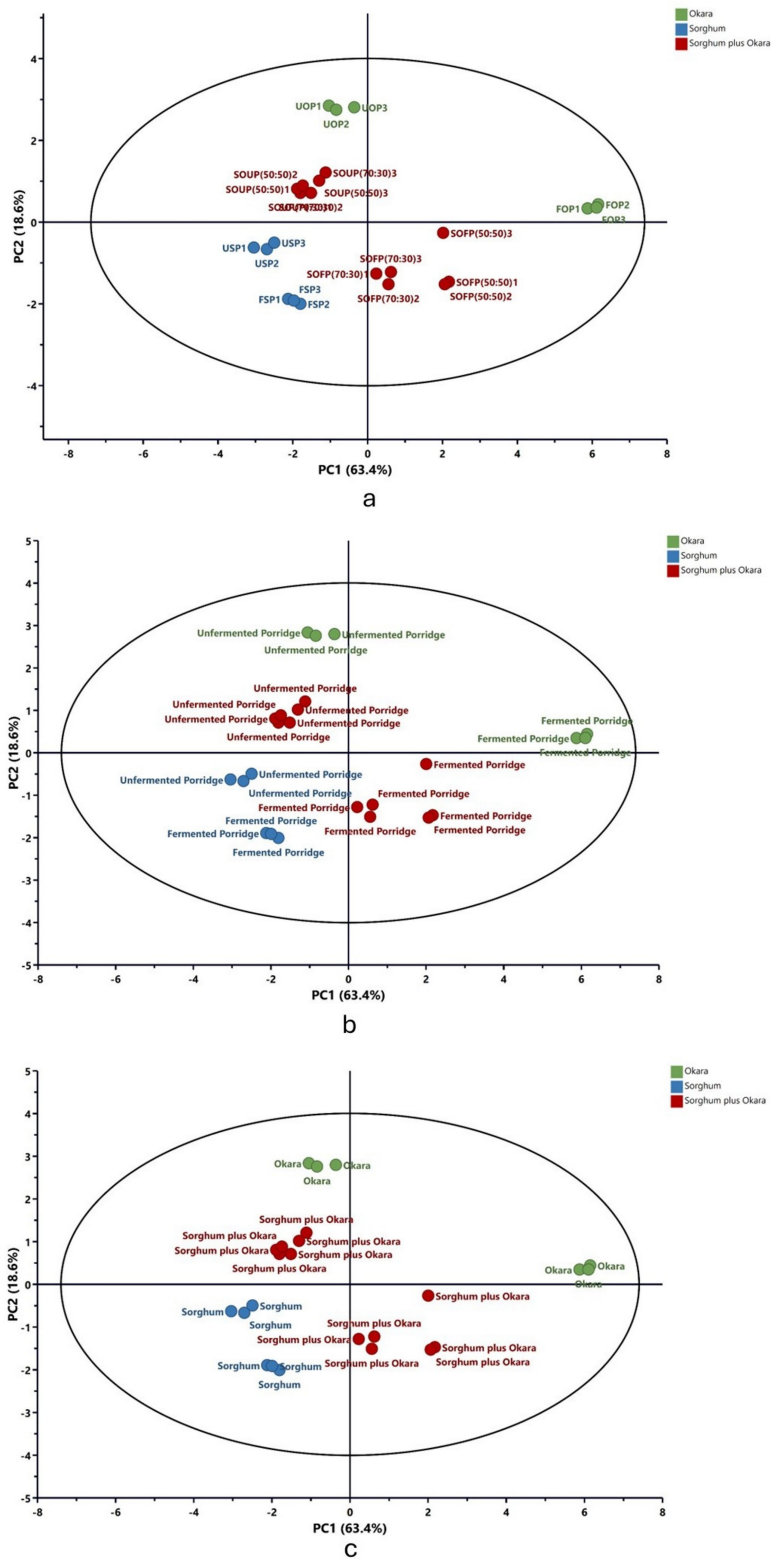
Principal component analysis (PCA) of mineral composition and bioaccessibility. USP, unfermented sorghum porridge; UOP, unfermented okara porridge; SOUP, sorghum okra unfermented porridge; FSP, fermented sorghum porridge; FOP, fermented okara porridge; SOFP, sorghum okara fermented porridge.

To better understand how the various porridge blends differ in their mineral profiles, specifically for iron, zinc, calcium, magnesium, phosphorus, and copper, as well as in the bioaccessibility of these minerals, PCA was conducted (Figure 1). The analysis revealed that the first two principal components, PC1 and PC2, accounted for a combined 82.0% of total variability, with PC1 contributing 63.4% and PC2 contributing 18.6%. Such a high cumulative percentage suggests that the main patterns in the mineral and bioaccessibility data are captured by these components, leading to a clear visual distinction among the porridge samples (Figure 1).

The PCA clearly illustrated that both flour composition and fermentation status shaped mineral amounts and bioavailability in sorghum porridges enriched with okara. In Figure 1a, fermented and unfermented sorghum-based porridges formed a separate cluster from the okara-based porridges, capturing the mineral disparity between the base materials (Table 1). The sorghum-okara combinational porridges occupy an intermediate location, confirming that mineral profiles are indeed dragged toward that of okara as the okara fraction rises. Thus, okara supplementation not only diversifies the mineral profile of sorghum porridges but also enhances the bioaccessibility of certain minerals (Table 2). Fermentation status emerged as a strong discriminating factor across all formulations. As shown in Figures 1a,b, fermented porridges were separated from unfermented porridges along PC1 (Figure 1b), irrespective of flour type, indicating that fermentation exerts a unifying effect on mineral solubility and bioaccessibility. The clustering pattern highlights the role of lactic acid fermentation in degrading phytates and other antinutritional factors, which in turn enhances the release and absorption of bound minerals. This agrees with studies reporting improved bioaccessibility of iron and zinc following fermentation of cereal-based porridges (7, 29, 30). Furthermore, the results indicated that the impact of fermentation on enhancing mineral content was more pronounced in okara-based porridges and their blends than in sorghum-only porridges. As illustrated in Figure 1a, fermented and unfermented samples of okara and flour blends were positioned in distinct quadrants, whereas both fermented and unfermented sorghum-only porridges clustered within the same quadrant with only slight separation. This difference may be attributed to variations in the food matrix. Interestingly, while fermentation reduced variability between sorghum- and okara-based formulations, the underlying differences in raw material composition remained evident. This suggests a synergistic interaction in which fermentation enhances overall mineral availability, but the flour type still determines the magnitude and balance of specific mineral contributions.

Figure 1c’s PCA analysis illustrates how flour type decisively shapes the mineral profile. Porridges made solely from sorghum align on the negative axis of PC1, while those with okara alone fall to the positive end; the mixed-grain porridges occupy an intermediate zone. Notably, the mixed samples lie closer to the sorghum cloud, yet rising okara percentages gradually nudge the patterns toward the okara side, reinforcing the dominance of okara’s mineral signatures. Overall, the data show that sorghum-okara combinations act as complementary mineral reservoirs. Fermentation, regardless of the starting blend, consistently boosts mineral bioaccessibility, validating the process as an effective step toward enhancing the nutrient density of cereal-legume products. These observations endorse the proposal that okara fortified, fermented sorghum gruels qualify as functional foods, offering improved mineral bioaccessibility and contributing to international priorities of mitigating mineral deficiencies through low-cost and sustainable dietary solutions.

### 3.4 Scanning electron microscopy

**Figure 2:**
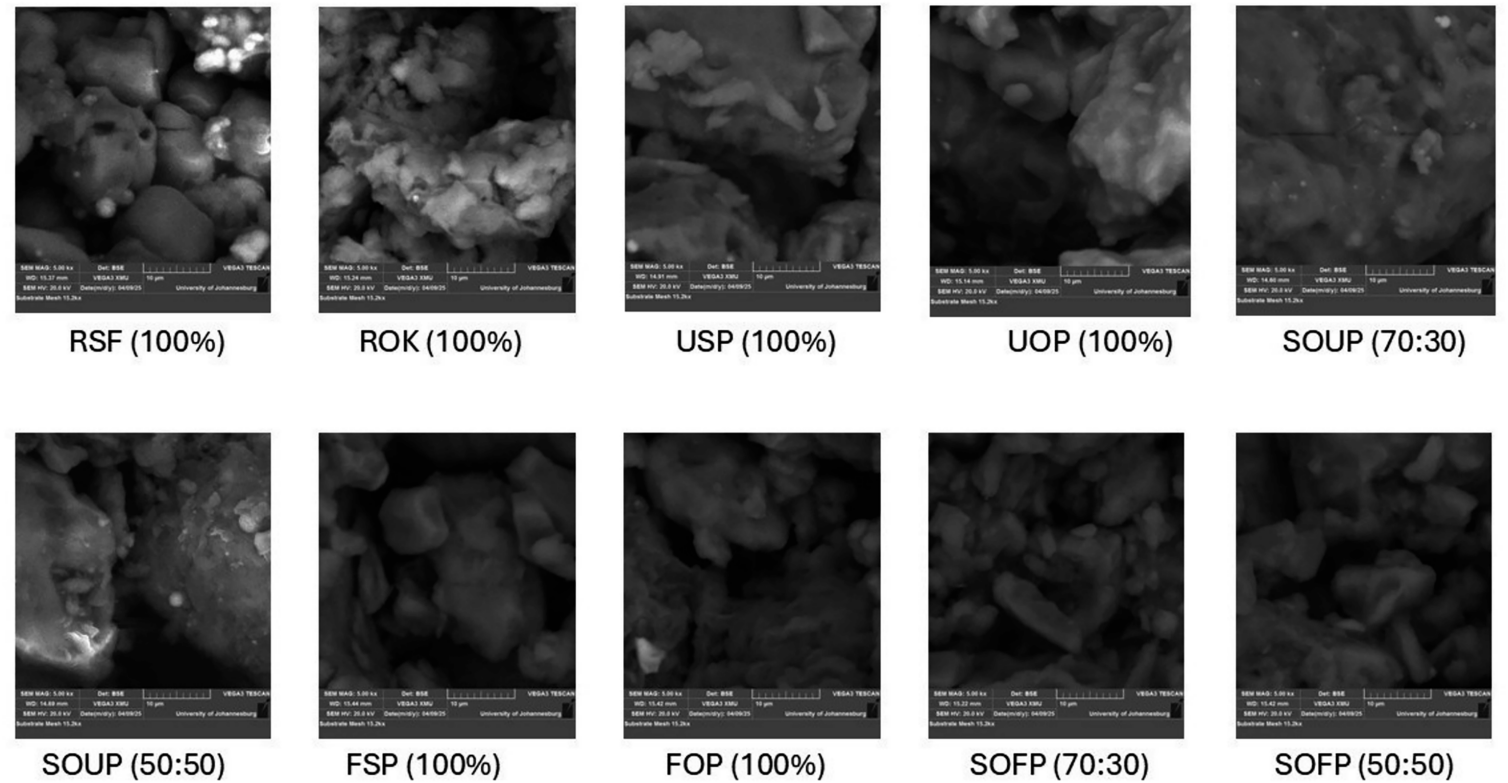
Scanning electron micrographs of raw sorghum flour, raw okara flour, and their porridges. RSF, raw sorghum flour; ROF, raw okara flour; USP, unfermented sorghum porridge; UOP, unfermented okara porridge; SOUP, sorghum okra unfermented porridge; FSP, fermented sorghum porridge; FOP, fermented okara porridge; SOFP, sorghum okara fermented porridge.

The microstructural features of raw flours, unfermented and fermented porridges derived from sorghum and okara, and their blends are presented in Figure 2. The SEM images shed light on the morphological changes that accompany compositional differences and processing, such as cooking and fermentation.

The raw sorghum flour had a fairly smooth surface, well-defined, compact polygonal starch granules with well-defined edges, and was characteristic of native starch granules (31). These granules, which are small and large, appeared distinct and varied a little in size. The raw okara flour, on the other hand, displayed irregular, amorphous agglomerates with a fibrous, flaky structure and no observable starch granules like sorghum, which agrees with an earlier report (32). This kind of morphology aligns with the renowned high fiber and protein content of okara, which lacks the ordered crystalline architecture that is characteristic of starchy materials (33).

The native granular structure was significantly altered during the thermal processing. In unfermented sorghum porridge, the distinct boundaries of starch granules metamorphosed into a swollen and collapsed granule dense amorphous matrix, indicating gelatinization (31). On the other hand, unfermented okara porridge still showed disorder, fibrous and semi-elastic shapes that were more cohesive compared to raw okara flour, likely due to partial hydration of proteins and subsequent denaturation. Blended unfermented porridges, a heterogeneous blend of sorghum:okara (70:30) unfermented porridge and sorghum:okara (50:50) unfermented porridge, showed more heterogeneous features. In the sorghum:okara (70:30) formulation, the matrix appeared dense and compact, with smoother surfaces compared to the raw materials, indicating the presence of partially swollen and disrupted starch granules embedded within the fibrous matrix of okara. Sorghum:okara (50:50) fermented porridge was visibly disordered in morphology with more pronounced amorphous areas. This was caused by the increased volume of okara, which interfered with the starch network formation, thereby lowering the visibility of the gelatinized granules as observed in the 70:30 formulation.

Fermentation further altered the microstructure of the porridge samples. The matrix of fermented sorghum porridge, for example, was more porous and exhibited a more open structure than the less porous and denser structure of unfermented sorghum porridge. The surface showed partial disintegration of the starch-protein matrix, which created loose structures, likely due to microbial enzymes that hydrolyze polysaccharide materials within the walls of the cells and some of the starch, creating the pores.

Fermented okara porridge (FOP) maintained its fibrous and irregular morphology, though slight surface erosion and increased porosity were observed, indicating microbial breakdown of protein and fiber constituents. This suggests limited but noticeable structural modifications during fermentation. For the fermented composite porridges, a highly disordered, porous microstructure was obvious. Sorghum:okara (70:30) displayed a loose, fragmented matrix with increased surface roughness, which indicates considerable enzymatic hydrolysis and relaxation of the matrix. Sorghum:okara (50:50) had the most disrupted structure, displaying a sponge-like amorphous shape with indistinguishable granular remnants. This suggests considerable alteration of the starch-protein-fiber complex, which could have implications for textural attributes and digestibility.

### 3.5 X-ray diffraction

**Figure 3:**
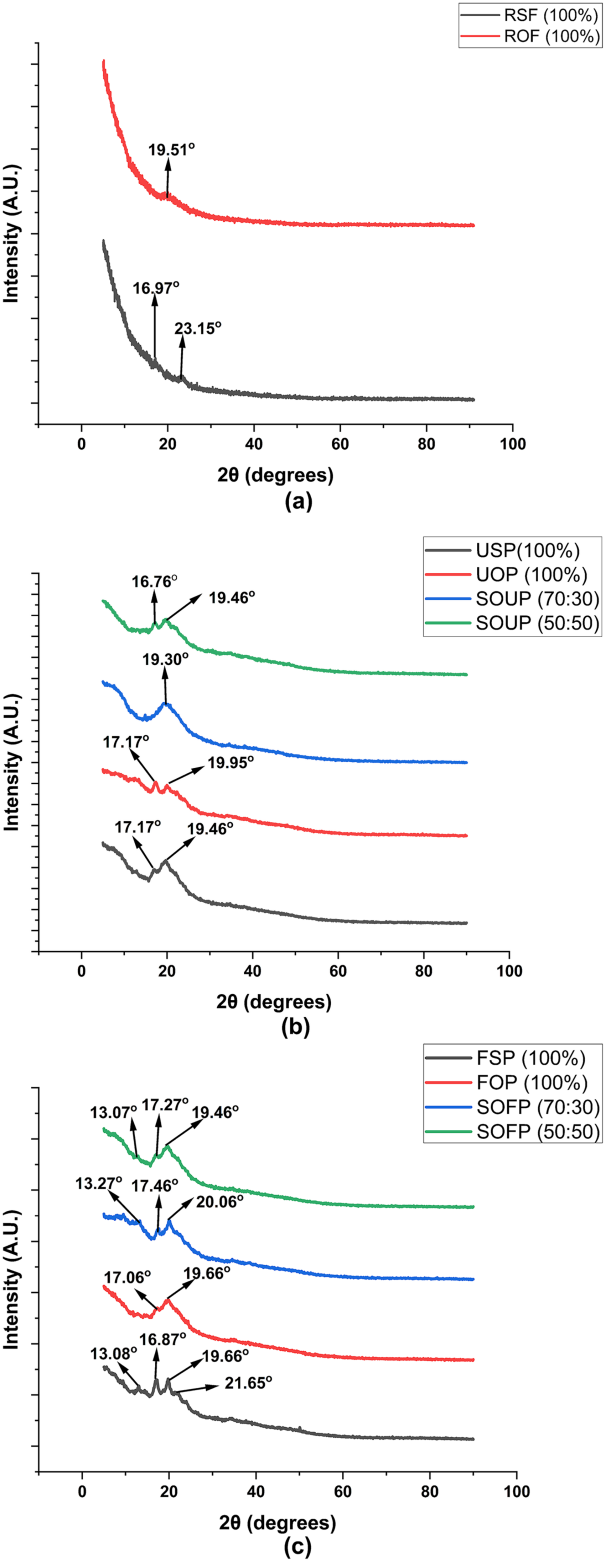
X-ray diffraction (XRD) spectra of raw flours **(a)**, unfermented porridges **(b)**, and fermented porridges **(c)** from sorghum and okara. RSF, raw sorghum flour; ROF, raw okara flour; USP, unfermented sorghum porridge; UOP, unfermented okara porridge; SOUP, sorghum okra unfermented porridge; FSP, fermented sorghum porridge; FOP, fermented okara porridge; SOFP, sorghum okara fermented porridges.

The X-ray diffraction (XRD) patterns obtained for the raw flours, unfermented porridges, and fermented porridges from sorghum and okara composite flours (Figure 3) revealed distinct differences in crystallinity and characteristic diffraction peaks, which are indicative of structural changes occurring during processing.

The raw sorghum flour showed peaks at 2θ 16.97 and 23.15°, which correspond to the A-type crystalline polymorph usually found in the starch of cereals (34, 35). The A-type pattern is indicative of a monoclinic unit cell with a framework of double helical chains, alongside a packed crystalline array commonly found in sorghum, maize, and wheat starches (36). On the other hand, the raw okara flour demonstrated a peak at 2θ = 19.51°, which is indicative of more amorphous or less crystalline and more disordered structures predominating (37). This is consistent with previously reported data suggesting that okara, which is the residue from soybeans, is composed of low starch and more dietary fiber and protein, which are normally amorphous, leading to weak XRD peaks (38).

When these materials were processed into porridges, distinct changes as well as diminutions of intensity of the diffraction peaks were observed. Both unfermented 100% sorghum porridge and unfermented 100% okara porridge showed retention of peaks at 2θ = 17.17° and 19.46° for the former and 17.17° and 19.95° for the latter. The emergence of the peaks within this range suggests that some crystalline structures are preserved, most likely due to the retrogradation of starches during cooling (39). The sorghum-okara unfermented composites also displayed characteristic peaks at 19.30° for the 70:30 blend and 16.76° and 19.46° for the 50:50 blend. The reason for the presence of these peaks might be the influence of okara’s non-starch polysaccharides, which may disrupt starch crystallization during gelatinization and cooling, and therefore, change the diffraction pattern. The decrease in the overall crystallinity in relation to the raw flours is in alignment with the partial gelatinization of the starch granules during the cooking process and the emergence of amorphous areas as water breaks down the crystalline lattices (39).

Changes were more pronounced in the fermented porridges. Fermented 100% sorghum porridge exhibited multiple peaks at 13.08°, 16.87°, 19.66°, and 21.65°, suggesting additional disruption and possible reorganization of starch granules. The most interesting peak to note is 13.08°, which could represent the development of V-type crystalline regions common in amylose-lipid complexes (39). These complexes could result from fermentation processes in which starch breakdown and lipid interactions can lead to such reorganization. The sorghum-okara fermented porridges showed significant peaks at 13.27°, 17.46°, and 20.06° (70:30) and 13.07°, 17.27°, and 19.46° (50:50). The observed diffraction peaks indicate that these composites still retain a certain degree of crystallinity, suggesting that structural order is still retained to some extent. This may result from a combination of retrogradation of starch from sorghum and the fibrous and proteinaceous okara matrix that influenced the structure during fermentation. Also, the appearance of an additional diffraction peak at approximately 2θ = 13° is indicative of the development of V-type crystalline regions, which are typically formed by starch-lipid or starch-protein complexes that are produced during the fermentation and cooking processes (39, 40).

These findings indicate that both fermentation and compositing with okara notably change the crystalline structure of sorghum-based porridges. The noted decreases and alterations in characteristic diffraction peaks imply significant changes regarding crystallinity and the formation of new crystalline complexes. Such changes are important because they affect the functional and nutritional attributes of the porridges strongly, such as digestibility, texture, and potential for resistant starch formation. Resistant starch with V-type crystallinity is valued for its health benefits, including enhanced glycemic control and improved colonic health.

### 3.6 Fourier transform infrared spectroscopy

**Figure 4:**
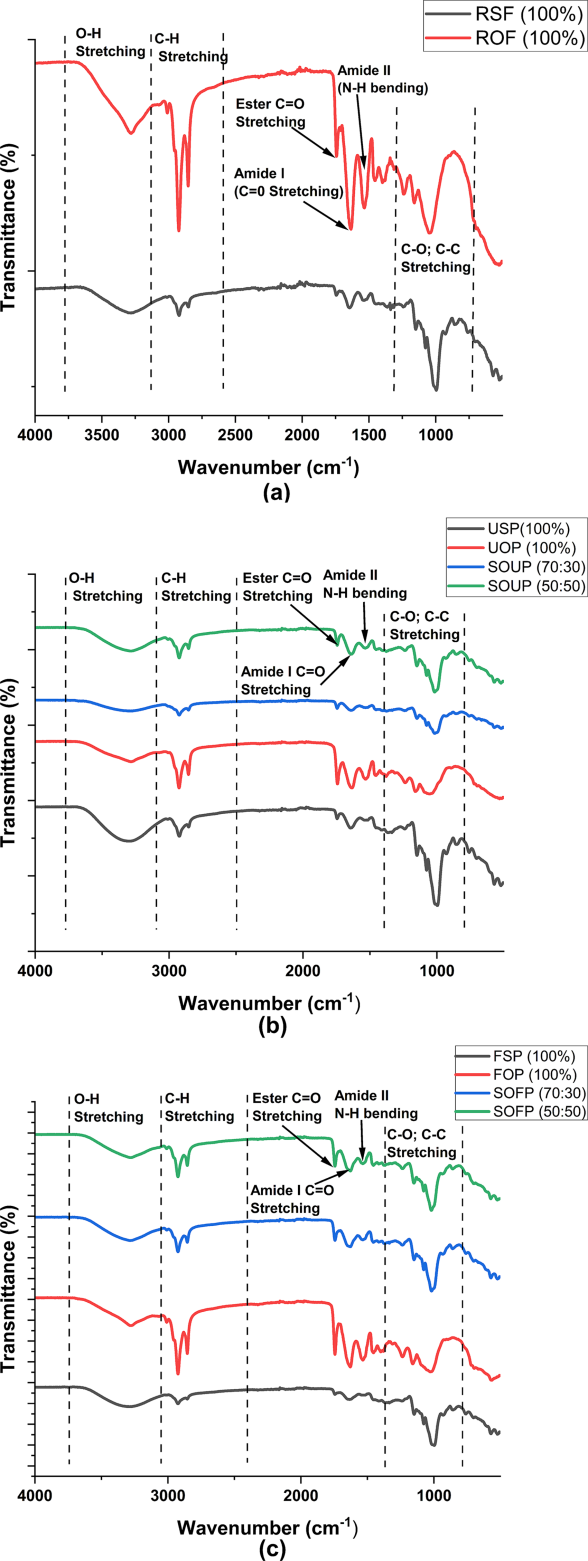
Fourier-transform infrared (FTIR) spectra of raw flours **(a)**, unfermented porridges **(b)**, and fermented porridges **(c)** from sorghum and okara. RSF, raw sorghum flour; ROF, raw okara flour; USP, unfermented sorghum porridge; UOP, unfermented okara porridge; SOUP, sorghum okra unfermented porridge; FSP, fermented sorghum porridge; FOP, fermented okara porridge; SOFP, sorghum okara fermented porridges.

The FTIR spectra of raw sorghum flour and raw okara flour (Figure 4a) exhibited characteristic absorption bands corresponding to major functional groups of carbohydrates, proteins, phenolics, and lipids. It was observed that both flours had broad absorption bands in the range of 3,290 cm^−1^ to 3,280 cm^–1^. This is characteristic of stretching vibrations of the –OH bonds commonly found in water, proteins, polysaccharides, and phenolic compounds (41, 42). This portion of the spectra was characterized by rough boundaries, with raw okara flour having a more intense peak, which may be due to more proteins than sorghum flour. Peaks at 2,922 cm^−1^ and 2,850 cm^−1^ suggest the C–H stretching vibrations of the aliphatic –CH₂ functional groups, which are common in lipids and proteins (32). Both the okara and sorghum flours had an absorption band at 1,745 cm^−1^, which can be considered as the C=O stretching of the ester bond carbonyl groups, which are found in lipids, uronic acids, and acetylated hemicellulose residues of fiber (38, 43, 44). The observed greater intensity of this peak in okara flour versus sorghum flour suggests that sorghum flour contained fewer ester-linked components and that okara flour had higher levels of lipids, along with acetylated hemicelluloses linked to its fibrous constituents. The amide I and amide II bands, approximately 1,642 cm^−1^ and 1,513 cm^−1^, associated with C=O stretching and N–H bending of proteins, respectively, were also stronger in raw okara flour, which aligns with its higher protein content (33, 41). Furthermore, the peaks at approximately 1,152 cm^−1^ and 1,001 cm^−1^, which are due to C–O and C–C bonds stretching in the polysaccharides, suggest that the starch and cellulose components are present (45).

The unfermented porridges (Figure 4b) showed similar absorption patterns, albeit with some differences in intensity and minor peak shifts. For example, the intensity reduction observed for the O–H stretching region at approximately 3,290 cm^−1^ in sorghum:okara unfermented porridge formulations relative to unfermented okara porridge indicates some interaction of sorghum with okara components, perhaps via hydrogen bonds. Similarly, the ester (C=O) peak at approximately 1,745 cm^−1^, though present in the composite porridges, was less intense compared to the porridge prepared solely from okara. The reduction in the intensity may be attributed to partial hydrolysis during porridge preparation and dilution by sorghum addition. Amide I and II peaks were present in all the porridge samples, which showed that proteins were present. There were some noticeable changes in the FTIR spectra of the fermented samples, as shown in Figure 4c. A marked decrease in the intensity of the ester C=O peak (~1,745 cm^−1^) was observed in fermented samples, particularly in the composite formulations and fermented sorghum porridge, indicating microbial lipolytic activity and degradation of esterified lipids. The peaks of amide I and II at 1,640 cm^−1^ and 1,515 cm^−1^, respectively, also showed weaker intensities, suggesting partial proteolysis of proteins by fermenting microbes. The peaks between 1,000 and 1,150 cm^−1^ were still visible, which indicates that some polysaccharides, such as starch and cellulose, still existed. However, some shifts could be seen, which might be the result of structural changes due to enzymatic processes during fermentation.

## 4 Conclusion

This study reveals how the incorporation of okara into sorghum, particularly at inclusion rates of 30 and 50% along with fermentation, greatly improves the instant porridge’s mineral composition and bioaccessibility. Fermentation, in addition to okara enrichment, enhanced the mineral composition, with the 50:50 fermented sorghum-okara porridge having the highest values of bioaccessible iron, zinc, magnesium, and phosphorus. SEM, XRD, and FTIR structural and compositional analyses evidenced profound fermentation-induced alterations that may enhance health-promoting properties. Collectively, these findings highlight the potential of integrating underutilized agro-industrial byproducts like okara into cereal-based food systems to enhance their nutritional and health-promoting attributes. Further studies should focus on comprehensive sensory analysis to evaluate consumer acceptability and overall satisfaction. This is instrumental in strategizing product refinement and in aiding the creation of commercially viable and public health functional porridge interventions formulated with nutrient-dense underutilized agro-industrial byproducts like okara.

## Data Availability

The raw data supporting the conclusions of this article will be made available by the authors, without undue reservation.
